# Modelling the Variability and the Anisotropic Behaviour of Crack Growth in SLM Ti-6Al-4V

**DOI:** 10.3390/ma14061400

**Published:** 2021-03-13

**Authors:** Rhys Jones, Calvin Rans, Athanasios P. Iliopoulos, John G. Michopoulos, Nam Phan, Daren Peng

**Affiliations:** 1 Centre of Expertise for Structural Mechanics, Department of Mechanical and Aerospace Engineering, Monash University, Clayton, VIC 3800, Australia; daren.peng@monash.edu; 2 ARC Industrial Transformation Training Centre on Surface Engineering for Advanced Materials, Faculty of Science, Engineering and Technology, Swinburne University of Technology, John Street, Hawthorn, VIC 3122, Australia; 3 Faculty of Aerospace Engineering, Delft University of Technology, Kluyverweg 1, 2629 HS Delft, The Netherlands; C.D.Rans@tudelft.nl; 4 Computational Multiphysics Systems Laboratory, Code 6394, Center for Materials Physics and Technology, US Naval Research Laboratory, Washington, DC 20375, USA; athanasios.iliopoulos@nrl.navy.mil (A.P.I.); john.michopoulos@nrl.navy.mil (J.G.M.); 5 Structures Division, Naval Air Systems Command, Patuxent River, MD 20670, USA; nam.phan@navy.mil

**Keywords:** additive manufacture, SLM Ti-6Al-4V, variability, anisotropy, fatigue crack growth

## Abstract

The United States Air Force (USAF) Guidelines for the Durability and Damage Tolerance (DADT) certification of Additive Manufactured (AM) parts states that the most difficult challenge for the certification of an AM part is to establish an accurate prediction of its DADT. How to address this challenge is the focus of the present paper. To this end this paper examines the variability in crack growth in tests on additively manufactured (AM) Ti-6Al-4V specimens built using selective layer melting (SLM). One series of tests analysed involves thirty single edge notch tension specimens with five build orientations and two different post heat treatments. The other test program analysed involved ASTM standard single edge notch specimens with three different build directions. The results of this study highlight the ability of the Hartman–Schijve crack growth equation to capture the variability and the anisotropic behaviour of crack growth in SLM Ti-6Al-4V. It is thus shown that, despite the large variability in crack growth, the intrinsic crack growth equation remains unchanged and that the variability and the anisotropic nature of crack growth in this test program is captured by allowing for changes in both the fatigue threshold and the cyclic fracture toughness.

## 1. Introduction

The regulatory requirements associated with additively manufactured (AM) parts for both civil and military aircraft are summarised in [1,2,3]. As noted in [3,4], and in the United Staes Air Force (USAF) airworthiness certification standard MIL-STD-1530D [5], it is essential that the variability in the crack growth rates be understood. This requirement is also highlighted in USAF Structures Bulletin EZ-19-01 [4], which specifically addresses the USAF guidelines for the durability and damage tolerance (DADT) certification of additively manufactured aircraft structural parts. Indeed, the ability to accurately assess the variability in crack growth is particularly important when performing the risk of failure analysis mandated in the US Joint Services Structural Guideline JSSG2006 [6]. As explained in Section 5.3 of MIL-STD-1530D [5] analysis is central to airworthiness certification, and the purpose of experimental tests is “to validate or correct analysis methods and results, and to demonstrate that requirements are achieved”.

The study by Virkler and Hillberry [7] is acknowledged to be the first paper to highlight the variability that can arise in the measured fatigue (long) crack growth rates (*da/dN*, where *a* is the crack length and *N* is the number of cycles) in conventionally manufactured metals. Whilst the paper by Iliopoulos et al. [8] highlighted the extensive variability that is associated with long cracks in Ti-6Al-4V built using a number of different AM processes, the paper by Rans et al. [9], which presented the *da/dN* versus Δ*K* curves associated with thirty Ti-6Al-4V single edge notch tension (SENT) specimens built using selective laser melt (SLM), is arguably the first to present a similar in depth study to that of Virkler where attention was focused on a single AM process. Unfortunately, as explained in [8], the expression used in [9] to determine the range in the stress intensity factor in a load cycle (Δ*K* = *K*_max_ − *K*_min_, where *K*_max_ and *K*_min_, are the maximum and minimum values of the stress intensity factor in a cycle) was inaccurate. Whilst correct expression for Δ*K* was given in [8] the corresponding *da/dN* versus Δ*K* were not. Consequently, one of the primary purposes of this paper is to present the accurate curves associated with these thirty SLM Ti-6Al-4V single edge notch tension (SENT) specimens and thereby highlight the extent of the variability associated with crack growth in SLM Ti-6Al-4V.

The paper by Molent and Jones [10] was the first to reveal that the variability in the *da/dN* versus Δ*K* curves given in [7] could be captured by allowing for the variability in the fatigue threshold term Δ*K_thr_* in Equation (2) the Hartman–Schijve equation [11], viz.

*da*/*dN* = *D*(Δ*κ*)^*p*^ (1)

The terms *D* and *p* are constants, and Δ*κ* is the crack driving force as given by Schwalbe in [12], viz.

Δ*κ* = (Δ*K* − Δ*K_thr_*)/(1 − (*K*_max_/*A*))^1/2^ (2)

where the term *A* is the cyclic fracture toughness. It has subsequently been shown [8,13,14,15,16,17,18] that the variability in crack growth in AM materials can often be accounted for by allowing for the variability in the fatigue threshold term Δ*K_thr_* and the cyclic fracture toughness term (*A*).

To address the main issue of accurately predicting the DADT, this paper also focuses in evaluating if this formulation can also be used to account for the variability in the *da/dN* versus Δ*K* curves presented by Ran’s et al. in [9]. The outcome of this initial study is that when *da/dN* is plotted as a function of Δ*κ* then each of these thirty curves essentially collapse onto the same master curve obtained for the growth of both long and small cracks in conventionally manufacture Ti-6Al-4V. It should be stressed that, this seminal finding represents the first time that any fracture mechanics-based study has been shown to be able to capture the underlying response in such a large cross section of tests on AM specimens built using a single AM facility. This example is particularly important given that MIL-STD-1530D mandates the use of fracture mechanics-based analyses in the certification process and that USAF Structures Bulletin EZ-19-01 states that the most difficult challenge for AM structural is to establish an “accurate prediction of structural performance” specific to DADT.

This study is complemented by a subsequent investigation into the ability of Equations (1) and (2) to capture the anisotropic behaviour of crack growth in ASTM compact tension (CT) SLM Ti-6Al-4V specimens. As such the studies presented in this paper illustrate how to address the challenge delineated in Structures Bulletin EZ-19-01, namely how to allow for the variability seen in crack growth in AM parts.

## 2. Materials and Methods

The data analysed in the present paper are taken either journals that are both peer reviewed and publicly available, refereed Conferences and texts that are publicly available (ISBN numbers are given in the associated reference), or from Google searches. Of these references ten are SCOPUS listed Journals, and five are available on various US government websites. The Book Chapters and Books referenced can all be found listed in SCOPUS, one reference can be found on the FAA website. The keywords used to find the references: Additive manufacturing, durability, damage tolerance, variability, and Hartman–Schijve. The exception to this is [2] which was presented at the Proceedings Indian Structural Integrity Society, 3rd Structural Integrity Conference and Exhibition (SICE), IIT, Mumbai, India, 11 December 2020 and which is not as yet available online.

## 3. Modelling the Variability in SLM TI-6AL-4V

Let us first examine the variability in the *da/dN* versus Δ*K* curves given in [9] for crack growth in SLMTi-6Al-4V. In this study thirty *R* = 0.1 tests were performed on single edge notch tension (SENT). The specimens tested had five different build orientations (0, 30, 45, 60 and, 90 degrees), and two different post heat treatments, namely: (a) specimens annealed at 735 °C and (b) specimens annealed at 735 °C and then subjected to hot isostatic pressing (HIP) for two hours. In [9] the build direction was defined relative to the crack in the SENT specimen. (By this it is meant that a build direction of 90 degrees corresponds to the case when the crack was at nright angle to the build direction.) Details of the processes, and the specimen identifiers are given in Table 1.

**Figure 1:**
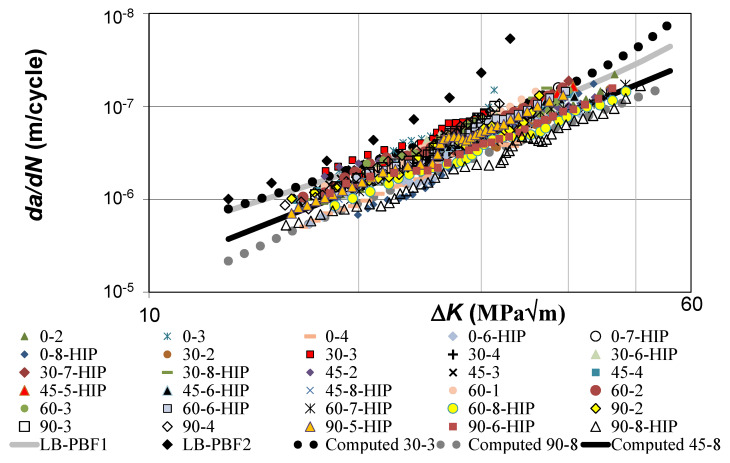
Variability in the thirty SLM Ti-6Al-4V tests reported in [9].

**Figure 2:**
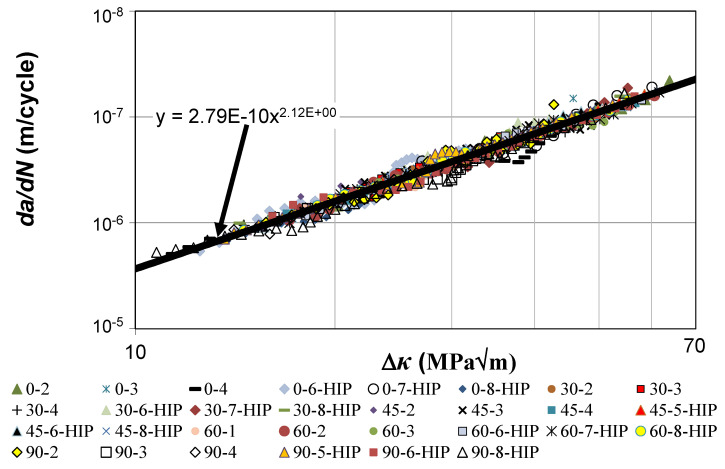
Hartman–Schijve representation of the thirty SLM Ti-6Al-4V tests.

The variability in the thirty *da/dN* versus Δ*K* curves is shown in Figure 1. (As noted in [8] the *da/dN* versus Δ*K* curves given in [9] were incorrect since the expression used to determine Δ*K* was incorrect. Whilst this error was corrected in [8], only a few selected *da/dN* versus Δ*K* curves were given.) Figure 2 reveals that the *da/dN* versus Δ*K* curves are largely bounded above by that of specimen 30-3, and below by specimen 90-8, which is HIPed. For comparison Figure 1 also contains the *R* = −1 *da/dN* versus Δ*K* curve determined in [17] for the growth of a short surface crack in an AM Ti-6Al-4V cylindrical specimen, that was fabricated using an M290 Laser Beam Powder Bed Fusion (LB-PBF) facility, subjected to constant amplitude loading with a maximum stress of 910 MPa. This curve is labelled LB-PBF1. The cyclic fracture toughness (*A*) of the material in this test was approximately 85 MPa √m, and Δ*K_thr_* was approximately 0.1 MPa √m, see [17]. Figure 1 reveals that the crack growth rate in specimen 30-3 is similar to the growth rate seen by the surface crack in specimen LB-PBF1.

To further illustrate the variability associated with AM Ti-6Al-4V Figure 1 also contains the *R* = −1 *da/dN* versus Δ*K* curve determined in [17] for a short surface crack in a specimen built using a Renishaw AM250 LB-PBF machine. In this instance the maximum applied stress was 268 MPa. This curve is labelled LB-PBF2. The cyclic fracture toughness (*A*) of the material in this test was found to be approximately 37 MPa √m and the fatigue threshold term (Δ*K_thr_*) was approximately 0.1 MPa √m, see [17]. Here, it should be noted that [17] explained that the difference in the crack growth rates in these two LB-PBF tests was primarily due to the differences in the cyclic fracture toughness’s.

Figure 2 reveals that if the curves shown in Figure 1 are plotted with *da/dN* as a function of Δ*κ*, then (allowing experimental error) the scatter in these thirty curves essentially vanishes. Figure 2 also reveals that these thirty tests lie on the same *da/dN* versus Δ*κ* curve determined for both long and short cracks in conventionally and AM Ti-6Al-4V, viz.

*da/dN* = 2.79 × 10^−10^ [(Δ*K* − Δ*K*_thr_)/(1 − *K*_max_/*A*)^1/2^]^1.99^ (3)

The values of Δ*K_thr_* and *A* used in Figure 2, and the corresponding values of the coefficient of determination (*R*^2^) are given in Table 2. (The mean value of the coefficients of determination given in Table 2 is approximately 0.96.) Here, it should be recalled that as shown in [13] the ASTM definition of the fracture toughness (Δ*K_th_*), which is arbitrarily chosen to be the value of Δ*K* at a crack growth rate *da/dN* of 10^−10^ m/cycle [19], is related to Δ*K_thr_* is via the expression:

Δ*K*_th_ = Δ*K*_thr_ + 0.62 (4)

To further illustrate how the variability in these tests can be captured using Equation (2) Figure 1 also presents plots of the computed curves for specimens 30-3 and 90-8 as well as for specimen 45-8, which is HIPed and which represents a mid-range (in the context of the present study) crack growth curve. These computed curves were determined using Equation (2) together with the values of *A* and Δ*K_thr_* given in Table 2.

To help quantify the effect of the HIPing process Table 3 presents the mean and standard deviations associated with specimens both with and without HIPing. As in [13,14] we see that in contrast to conventionally manufactured specimens the variability associated with the cyclic fracture toughness (*A*) is quite large. The mean value of *A* for the annealed specimens of approximately 79 MPa √m is similar to the mean value of approximately 83 MPa √m associated with the forty different AM Ti-6Al-4V specimens analysed in [8,13]. The standard deviation associated with the SLM specimens is approximately 23 MPa √m. This value is lower than the value of 49 MPa √m obtained for the specimens analysed in [8,13]. This is to be expected since the later value covers specimens fabricated using a variety of different AM processes, viz. SLM, Direct Metal Deposition (DMLS), Laser Engineered Net Surface (LENS), etc., and includes specimens left as built, after annealing, and/or after HIPing. When specimens that were either left in the as fabricated state or HIPed are removed from the data being considered then the mean value increases slightly to approximately 89 MPa √m with a standard deviation of approximately 57 MPa √m.

Table 3 reveals that the mean value of *A* for the SLMS specimens that were both annealed and HIPed is approximately 96.6 MPa √m. At first glance this would suggest that HIPing is advantageous. However, the standard deviation associated with these two sets of SLM specimens is quite large, and hence caution is urged with respect to this observation.

Table 3 also reveals that the mean values of the fatigue threshold term Δ*K_thr_* associated with the annealed (only) and the annealed and HIPed specimens are 3.2 and 3.8 MPa √m, respectively. The corresponding standard deviations are 2.4 and 1.9 MPa √m. These values are comparable with the mean values of 3.3 and 3.5 MPa √m associated with all of the forty different AM Ti-6Al-4V specimens analysed in [8,13], and with the value obtained when specimens that either were left in the as fabricated state or HIPed are removed from the data being considered. However, the mean values are misleading in that in several of these tests the value of Δ*K_thr_* was significantly lower.

### Crack Growth in ASTM Compact Tension SLM Ti-6Al-4V Specimens as Function of Crack Orientation Relative to the Build Direction

**Figure 3:**
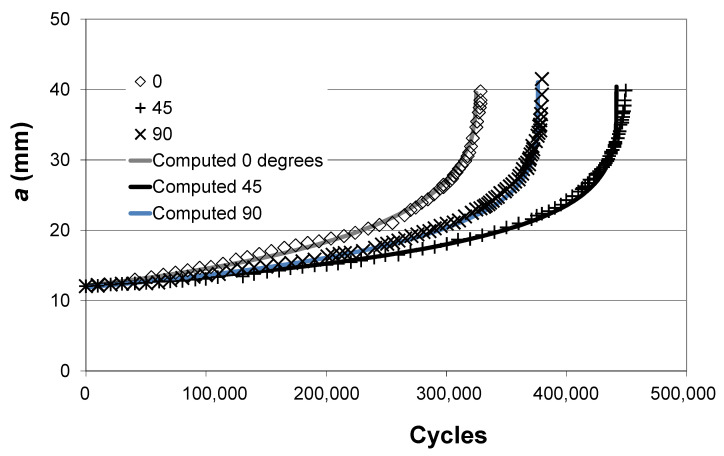
Measured and computed crack growth histories for the SLM Ti-6Al-4V tests reported in [20].

Let us next consider the *R* = 0.1 crack growth histories presented in [20] for crack growth in a 10 mm thick ASTM compact tension (CT) specimen with cracks at 0°, 45°, and 90° to the build direction in SLM Ti-6Al-4V. The measured and computed crack growth histories are shown in Figure 3 where we see excellent agreement. The values of Δ*K_thr_* and *A* used in Figure 3 are given in Table 4.

## 4. Implications for the Durability Analysis of AM Parts

**Figure 4:**
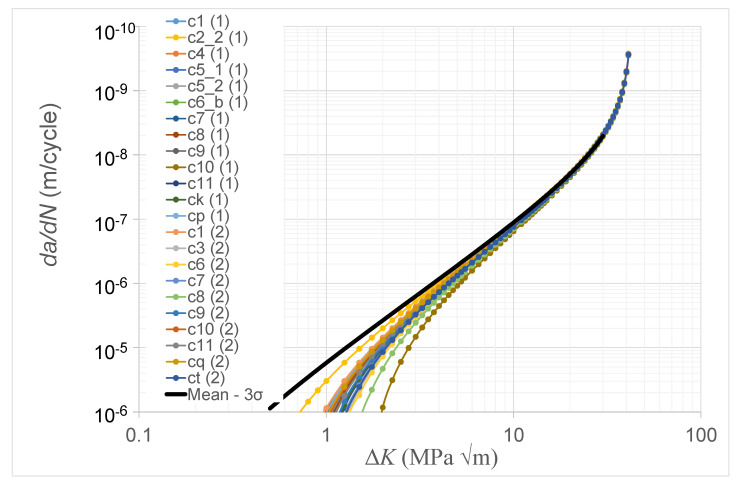
Variability in the crack growth curves for the twenty-three small surface breaking cracks in 7050-T7451 specimens [16]. The labelling convention used for these twenty-three cracks is taken from [16].

USAF Structures Bulletin EZ-19-01 [3] explains that durability analysis is essential to the certification of AM parts. Lincoln and Melliere [21], as part of the USAF F-15 program, and [11,14] have shown that a durability analysis necessitates the use of the associated small crack *da/dN* versus Δ*K* curve (a similar statement is contained in Appendix X3 of the ASTM fatigue test standard E647-15 [19]). Structures Bulletin EZ-19-01 [3] also requires the use of a minimum equivalent initial damage size (EIDS) of 0.254 mm (0.01 inch). (This value is taken from the Joint Services Structural Guidelines 2006 [3].) Whereas the paper by Virkler and Hillberry [7] is acknowledged to be first to illustrate the variability associated with long cracks in metals, the paper by Kundu et al. [16], which presented the crack growth histories associated with twenty three small surface breaking cracks with length scales of the order of 0.254 mm in AA7050-T7451 aluminium alloy specimens, was (to the best of the authors knowledge) the first to examine the variability in the growth of small surface breaking cracks with sizes comparable to that of the EIDS required in [3]. This study revealed that the variability in the crack growth histories was accurately captured allowing for variability in Δ*K_thr_*. The resultant variability in the *da/dN* versus Δ*K* curves associated with these twenty-three (small) surface breaking cracks is shown in Figure 4.

Of course, the variability seen in Figure 4 is associated with a limited data set, and as such it may not necessarily capture the extent of the true variability in the material properties. To account for such limited data sets Niu [22] and Rouchon [23] suggest adopting a statistical approach whereby the ‘A basis’ and ‘B basis’ properties are determined. An ‘A basis’ mechanical property value equals the mean value minus three standard deviations and is the value above which at least 99% of the population of values is expected to fall with a confidence of 95% [22]. A ‘B basis’ mechanical property value equals the mean value minus two standard deviations and is the value above which at least 95% of the population of values is expected to fall with a confidence of 95% [22]. The values of Δ*K_thr_* given in [16] for these twenty-three cracks are given in Table 5. The mean value of Δ*K_thr_* is approximately 0.80 MPa√m and the standard deviation (σ) is approximately 0.24 MPa√m. This yields a Mean- 3σ of approximately 0.1 MPa √m. This curve is also shown in Figure 4. Interestingly the Mean- 3σ curve shown in Figure 4 for these size EIDS is close to that given in [24,25,26] for the growth of “small” cracks from small near micron size surface discontinuities in 7050-T7451. It is also the same as the values determined in [17] for the growth of small surface breaking cracks in LPBF Ti-6Al-4V.

Taking the results of this study into the variability of near EIDS size surface breaking cracks into consideration, and noting that the fastest growing long crack in the SLM Ti-6Al-4V tests given in [9] can be approximated by using Equations (1) and (2) together with a low value for the fatigue threshold term Δ*K_thr_*, it is hypothesised that this phenomena, i.e., that the worst case curve associated with surface breaking cracks with dimensions as per the minimum allowable EIDS given in [3] for the durability analysis of an AM part would also resemble the corresponding small crack curve, may also hold for AM parts. However, testing is required to evaluate this hypothesis.

## 5. Conclusions

USAF Structures Bulletin EZ-19-01 states that the most difficult challenge for AM structural is to establish an “accurate prediction of structural performance” specific to DADT. It also notes the importance of being able to account for the variability in the crack growth rates associated with AM parts. To meet this challenge the present has examined the variability in the crack growth rates associated with two studies into crack growth in SLM Ti-6Al-4V. One of the studies analysed, involved thirty single edge notch tension specimens with five build orientations and two different post heat treatment methods. The other study involved ASTM standard CT specimens with three different build directions. The results of this analysis highlight the ability of the Hartman–Schijve crack growth equation to capture the variability and the anisotropic behaviour of crack growth in SLM Ti-6Al-4V. This seminal finding represents the first time that any fracture mechanics-based study has been shown to be able to capture the underlying response in such a large cross section of tests on AM specimens built using a single AM facility. This development is central to meeting the certification requirements delineated in MIL-STD-1530D and EZ-19-01.

It is also hypothesised that the worst-case curve associated with surface breaking cracks with dimensions as per the minimum allowable EIDS given in EZ-19-01 for the durability analysis of an AM part should resemble the corresponding small crack curve. However, additional testing is required to evaluate this hypothesis. This finding, once further validated, will have significant implications for the economic life/durability certification of AM parts.

## Figures and Tables

**Table 1:** Notation associated with the specimen tests reported in [9].

Build Angle	Treatment	Descriptor
0°	annealed at 735 °C	00-2
ibid	ibid	00-3
ibid	ibid	00-4
ibid	annealed at 735 °C and then HIPed for	00-6
ibid	2 h at 920 °C and 1000 bar	00-7
ibid	ibid	00-8
30°	annealed at 735 °C	30-2
ibid	ibid	30-3
ibid	ibid	30-4
ibid	annealed at 735 °C and then HIPed for 2 h at 920 °C 1000 bar	30-6
ibid	ibid	30-7
ibid	ibid	30-8
45°	annealed at 735 °C	45-2
ibid	ibid	45-3
ibid	ibid	45-4
ibid	annealed at 735 °C and then HIPed for	45-5
ibid	2 hrs 920 °C 1000 bar	45-6
ibid	ibid	45-8
60°	annealed at 735 °C	60-2
ibid	ibid	60-3
ibid	annealed at 735 °C and then HIPed for	60-6
ibid	2 hrs at 920 °C and 1000 bar	60-7
ibid	ibid	60-8
90°	annealed at 735 °C	90-2
ibid	ibid	90-3
ibid	ibid	90-4
ibid	annealed at 735 °C and then HIPed for 2 h 920 °C 1000 bar	90-5
ibid	ibid	90-6
ibid	ibid	90-8

**Table 2:** Values used in Figure 2.

Build Angle	Descriptor	∆*K_thr_* (MPa √m)	*A* (MPa √m)	Coefficient of Determination (*R^2^*)
0°	00-2	5.10	88.0	0.97
ibid	00-3	1.50	54.5	0.95
ibid	00-4	7.80	73.0	0.91
ibid	00-6 (HIPed)	5.40	107.0	0.96
ibid	00-7 (HIPed)	4.92	67.0	0.95
ibid	00-8 (HIPed)	8.20	70.0	0.99
30°	30-2	5.90	105.0	0.95
ibid	30-3	0.10	63.5	0.97
ibid	30-4	4.10	73.0	0.97
ibid	30-6 (HIPed)	1.30	85.0	0.91
ibid	30-7 (HIPed)	2.20	73.0	0.93
ibid	30-8 (HIPed)	2.55	65.0	0.97
45°	45-2	0.10	134.0	0.98
ibid	45-3	1.90	73.0	0.94
ibid	45-4	2.70	85.0	0.99
ibid	45-5 (HIPed)	1.50	76.0	0.99
ibid	45-6 (HIPed)	2.40	90.0	0.98
ibid	45-8 (HIPed)	3.10	128.0	0.99
ibid	60-1	3.00	61.0	0.99
ibid	60-2	1.90	74.0	0.99
60°	60-3	0.10	98.3	0.92
ibid	60-6 (HIPed)	3.70	70.0	0.99
ibid	60-7 (HIPed)	3.70	116.0	0.97
ibid	60-8 (HIPed)	5.00	140.0	0.99
ibid	90-2	1.95	93.8	0.85
90°	90-3	5.90	49.7	0.98
ibid	90-4	5.01	51.0	0.99
ibid	90-5 (HIPed)	4.00	80.0	0.98
ibid	90-6 (HIPed)	3.80	123	0.99
ibid	90-8 (HIPed)	6.20	168.0	0.96

**Table 3:** Mean and standard deviations of Δ*K_thr_* and *A* associated with SLM Ti-6Al-4V specimens with and without HIPing.

	Mean Value	Standard Deviation
Annealed at 735 °C		
*A* (MPa × √m)	78.6	23.0
Δ*K_thr_* (MPa × √m)	3.2	2.4
Annealed at 735 °C and then HIPed for 2 hrs at 920 °C 1000 bar		
*A* (MPa × √m)	96.6	31.6
Δ*K_thr_* (MPa × √m)	3.8	1.9

**Table 4:** Values used in Figure 3.

Build Direction	∆*K_thr_* (MPa × √m)	*A* (MPa √m)
0°	2.0	71
45°	3.8	52
90°	3.15	48.5

**Table 5:** Values of the term Δ*K_thr_* given in [16].

Crack Descriptor	∆*K_thr_* (MPa √m)
c1	0.7
c2_2	0.35
c4	0.75
c5_1	0.8
c5_2	0.6
c6_b	0.75
c7	0.85
c8	0.72
c9	0.65
c10	1.6
c11	0.61
ck	0.83
cp	0.63
c1	0.6
c3	0.95
c6	1
c7	0.9
c8	1.2
c9	0.8
c10	0.68
c11	0.75
cq	0.66

## Data Availability

Data sharing is not applicable to this article.
